# *vanB*-Gene-Dominated Resistance in *Enterococcus* spp. and Silent *vanA*-Gene Carriage in Phenotypically Susceptible Isolates: Genomic Epidemiology in Two Hospitals in Latvia

**DOI:** 10.3390/antibiotics15060601

**Published:** 2026-06-12

**Authors:** Inga Mauliņa, Linda Labecka, Aivars Cīrulis, Juris Ķibilds, Renārs Erts, Evija Bebre, Barba Vilima, Karīna Ortlova, Antoņina Muižzemniece, Elvīra Lavrinoviča, Dace Rudzīte, Indra Zeltiņa, Dace Bandere, Angelika Krūmiņa

**Affiliations:** 1 Vidzeme Hospital, Jumaras Street 195, LV-4201 Valmiera, Latvia; barba.vilima@vidzemesslimnica.lv (B.V.); karina.ortlova@vidzemesslimnica.lv (K.O.); antonina.muizzemniece@vidzemesslimnica.lv (A.M.); 2 Riga East Clinical University Hospital, Hipokrata Street 2, LV-1038 Riga, Latvia; elivra.lavrinovica@aslimnica.lv (E.L.); dace.rudzite@aslimnica.lv (D.R.); indra.zeltina@aslimnica.lv (I.Z.); 3 Department of Applied Pharmacy, Riga Stradiņš University, Konsula Street 21, LV-1007 Riga, Latvia; dace.bandere@rsu.lv; 4 Institute of Food Safety, Animal Health and Environment “BIOR”, Lejupes Street 3, LV-1076 Riga, Latvia; linda.labecka@bior.lv (L.L.); aivars.cirulis@niri.lv (A.C.); juris.kibilds@bior.lv (J.Ķ.); evija.bebre@bior.lv (E.B.); angelika.krumina@rsu.lv (A.K.); 5 Faculty of Medicine and Life Sciences, University of Latvia, Jelgavas Street 3, LV-1004 Riga, Latvia; renars.erts@lu.lv; 6 National Institute of Research and Innovation, Ratsupites Street 1, LV-1067 Riga, Latvia; 7 Department of Infectology, Riga Stradiņš University, Dzirciema Street 16, LV-1007 Riga, Latvia; 8 Baltic Biomaterials Centre of Excellence, Headquarters at Riga Technical University, Paula Valdena Street 3, LV-1048 Riga, Latvia

**Keywords:** *Enterococcus*, *vanA*, *vanB*, vancomycin-variable *Enterococcus*, whole-genome sequencing, cgMLST, ST80, ST78, ST6

## Abstract

Background/Objectives: Vancomycin-resistant (VRE) and vancomycin-variable (VVE) *Enterococcus* spp. represent an increasing clinical challenge due to limited treatment options and the potential for undetected dissemination of such resistance genes. Data on *Enterococci* genomic epidemiology in healthcare settings remain rather limited. Our study aimed to investigate vancomycin resistance determinants in *Enterococcus* spp., clonal structure, and occurrence of VVE using whole-genome sequencing (WGS) in Latvia. Methods: Clinical isolates collected from hospitalised patients in two tertiary-level hospitals in Latvia (2021–2024) were analysed using WGS following routine laboratory identification. Vancomycin resistance determinants were identified in silico, along with MLST and cgMLST genotyping. Results: Of 532 sequenced isolates, 482 met the quality and inclusion criteria. *E. faecalis* (56.64%) and *E. faecium* (40.25%) predominated. Among 125 isolates carrying vancomycin resistance genes, *vanB* (54.40%) was the most frequent, followed by *vanA* (38.20%) and *vanC* (6.40%); *vanC* was restricted to *E. gallinarum* and *E. casseliflavus*. Vancomycin resistance was more prevalent in *E. faecium* (51.03%) than in *E. faecalis* (6.59%). cgMLST identified outbreak clusters among *E. faecium* ST80 and ST78 with complex type-specific resistance patterns and hospital specificity. *E. faecalis* showed polyclonal endemicity with the *vanB* gene present in different clades. Three (0.62%) vancomycin-variable *E. faecium* (VVE) isolates were identified in one hospital, harbouring *vanA*-type gene clusters comprising *vanHAX* but lacking the sensory gene *vanS* and the regulatory gene *vanR*. Conclusions: The *VanB* gene predominated in both hospitals, driven by clonal expansion of hospital-adapted *E. faecium* ST80/ST78, contrasting with earlier *vanA* predominance in Europe but aligning with recent regional *vanB* trends. The detection of VVE highlights clinically relevant genotype–phenotype discordance, underscoring the importance of integrating genomic surveillance with routine phenotypic testing to detect cryptic resistance and guide effective antimicrobial therapy.

## 1. Introduction

Antimicrobial resistance (AMR) represents a massive global crisis with escalating momentum and dire future projections. According to the WHO, bacterial AMR was directly responsible for 1.27 million deaths worldwide in 2019. For the year 2050, this number might increase up to 10 million deaths [1,2]. In Europe, WHO/ECDC data from 2023 report >670,000 infections and 33,000 deaths attributable to resistant bacteria, while the US CDC estimates >2.8 million AMR infections and 35,000 deaths annually [3].

*Enterococcus* spp. are ubiquitous Gram-positive bacteria constituting part of the normal intestinal microbiota [4,5]. These organisms may cause severe infections if they acquire various virulence and resistance mechanisms and/or translocate to other sites in the organism, including normally sterile sites and body fluids [6,7]. They are frequently implicated in urinary tract infections (UTIs), bloodstream infections (BSIs), endocarditis, intra-abdominal infections, and device-associated infections, as supported by recent epidemiological reviews and studies [6,8].

More than 30 species have been described, but *Enterococcus faecalis* and *Enterococcus faecium* account for the most clinically relevant infections [4,9]. While *E. faecalis* remains a major clinical species, *E. faecium*, as a member of the ESKAPE (*Enterococcus faecium*, *Staphylococcus aureus*, *Klebsiella pneumoniae*, *Acinetobacter baumannii*, *Pseudomonas aeruginosa*, *Enterobacter* spp.) pathogen group, presents a particular challenge due to its pronounced antimicrobial resistance capacity, causing complex healthcare-associated infections and demonstrating potential for dissemination in the general population [2,9,10,11,12,13].

Vancomycin, a glycopeptide antibiotic, remains a cornerstone for treating various resistant infections; however, the global emergence of vancomycin-resistant *Enterococcus* spp. (VRE) has substantially limited therapeutic options [14,15]. In Europe, vancomycin resistance is primarily associated with *E. faecium* and is linked to increased morbidity, prolonged hospitalisation, and higher mortality [9,12,13,16,17,18].

Resistance to vancomycin is mediated by van gene clusters that modify the terminal peptides of peptidoglycan precursors, thereby reducing the binding affinity of glycopeptide antibiotics and leading to therapeutic failure [19]. Nine *van* gene clusters have been described (*vanA*, *vanB*, *vanC*, *vanD*, *vanE*, *vanG*, *vanL*, *vanM*, *vanN*), with *vanA* and *vanB* being the most clinically relevant acquired resistance determinants [20,21,22,23]. *VanA* confers high-level resistance to both vancomycin and teicoplanin, typically located in transferable transposons, while *vanB* mediates a variable resistance rate against vancomycin but preserves susceptibility against teicoplanin, often chromosomally integrated [23,24]. *VanC* represents intrinsic resistance primarily in *E. gallinarum* and *E. casseliflavus* [19,23].

Recent European studies indicate a gradual epidemiological shift from *vanA* towards *vanB*-mediated resistance in some regions, underscoring the need for local genomic surveillance [9,12,13,25]. Therefore, whole-genome sequencing (WGS), coupled with core genome multilocus sequence typing (cgMLST), has become essential for high-specificity surveillance and outbreak detection [26]. By analysing MLST data, long-term lineages (e.g., ST80 as a CC17 hospital clone over decades) can be observed, whereas cgMLST complex types represent outbreak-relevant transmission clusters (≤10–20 allele differences) [27,28]. Importantly, WGS facilitates the identification of vancomycin-variable *Enterococcus* spp. (VVE) isolates harbouring *van* genes (so-called “silent” *van* genes) that show phenotypic susceptibility to vancomycin [23], posing diagnostic and infection control challenges due to their potential to express resistance under selective pressure (e.g., during initiation of vancomycin therapy); VVE has been increasingly reported in several European countries, including the Netherlands, Denmark, and Italy [29,30,31,32,33]. In *van* gene clusters, inducible glycopeptide resistance is controlled by the *vanRS* two-component regulatory system [30,34,35]. *VanS* functions as a membrane-associated sensor histidine kinase that responds to glycopeptide-induced cell wall perturbations and undergoes autophosphorylation, subsequently transferring the phosphate group to the response regulator *vanR* [30,34,35]. *VanR* operates as a response regulator that activates transcription of *van* resistance genes. Phosphorylated *vanR* mediates transcriptional activation of resistance genes such as *vanHAX* [30,34,35]. While *vanR* may be activated independently of *vanS* via alternative phosphodonors, it remains essential for transcriptional activation of *van* resistance genes [30,34,35].

In Latvia, studies, except for one recent study [36], have largely relied on phenotypic susceptibility testing, providing limited insight into clonal structure, transmission, and *van* genes. To date, no data exist on the vancomycin-variable *Enterococcus* spp. (VVE) isolates harbouring *van* genes while remaining phenotypically susceptible to vancomycin, unlike in Denmark or other European countries [31,32,33,37,38].

The aim of this study was to investigate the molecular epidemiology of vancomycin-resistant and vancomycin-variable *Enterococcus* spp. isolated from hospitalised patients in two tertiary-level hospitals in Latvia using whole-genome sequencing (WGS). By integrating genomic resistance profiling and clonal distribution among various species within these hospitals, this study provides updated insights into the local epidemiology of VRE and VVE, identifies circulating *van* gene variants, and detects potential outbreak-associated clones [36]. The study also provides regional data on *van* genes and VVE to support surveillance and infection control strategies nationally, in the wider Northern European context, and more broadly across Europe [23,24,30,33,38,39].

## 2. Results

### 2.1. Study Population

A total of 532 *Enterococcus* isolates from both hospitals were sequenced, including 325 isolates from Riga East University Hospital (REUH) (61.1%) and 207 isolates from Vidzeme Hospital (VH) (38.9%).

After quality control of all sequenced samples, 19 isolates (REUH: *N* = 11; VH: *N* = 8) were excluded due to insufficient sequencing quality. Thus, 513 isolates (REUH: *N* = 314; VH: *N* = 199) were retained for further genomic analysis. An additional 31 isolates (REUH: *N* = 23; VH: *N* = 8) were excluded according to the study exclusion criteria, as they had been obtained from outpatients, including regular dialysis patients classified as ambulatory patients in Latvia. In total, 50 isolates were excluded from further analysis.

As a result, the final analysis included 482 isolates, of which 291 (60.37%) originated from REUH and 191 (39.63%) from VH. These isolates were obtained from 402 unique adult hospitalised patients and originated from routine diagnostics across different hospital settings at both hospitals.

The median age at hospitalisation was higher among women than men (73.0 years [Q1–Q3: 64.0–82.0] vs. 70.0 years [57.8–79.0], *p* < 0.01). When stratified by hospital, patients treated in VH were older than those treated in REUH within gender strata (75.0 years [65.0–82.0] vs. 69.0 years [50.2–79.0], *p* < 0.01). However, despite statistical significance, the magnitude of these differences was small, indicating substantial overlap in age distributions between groups, as shown in Table 1.

### 2.2. Distribution of Biological Specimens for Enterococcus spp. Investigation

Among the 482 *Enterococcus* spp. isolates, urine was the most common source (*N* = 240), followed by soft tissue specimens (*N* = 105). Blood cultures accounted for 44 isolates, and faecal samples, 42 isolates. Lower respiratory tract secretions and sterile body fluids contributed 22 isolates each. The distribution of biological specimen types, stratified by hospital, is presented in Table 2.

Among the 482 *Enterococcus* isolates, *Enterococcus faecalis* was the most frequently identified species (*N* = 273; 56.64%), followed by *Enterococcus faecium* (*N* = 194; 40.25%). Other species were identified infrequently, including *Enterococcus gallinarum* (*N* = 6; 1.24%), *Enterococcus avium* (*N* = 3; 0.62%), *Enterococcus casseliflavus* and *Enterococcus durans* (*N* = 2 each; 0.41%), and *Enterococcus hirae* and *Enterococcus raffinosus* (*N* = 1 each; 0.21%). A detailed list of all species and their distribution across both hospitals is presented in Table 3. The distribution of *Enterococcus* species according to biological specimen type is presented in Table 4.

### 2.3. Vancomycin Resistance Determinants

Vancomycin resistance-associated genes were identified in 125 isolates. In our study, we identified three *van* genes—*vanA*, *vanB*, and *vanC*. The *VanA* gene was identified in 49 (38.20%) isolates (*E. faecium*-48, *E. raffinosus*-1). The *VanB* gene was detected in 69 (54.40%) isolates (*E. faecium*-51, *E. faecalis*-18). The *VanC* gene was detected in eight (6.40%) isolates—six *E. gallinarum* and two *E. casseliflavus*.

**Figure 1:**
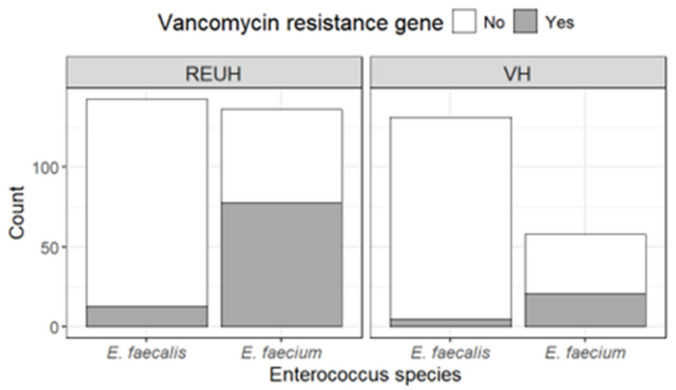
Positive and negative cases of vancomycin resistance genes in *E. faecalis* (13/129 and 5/126) and *E. faecium* (78/58 and 21/37) in REUH and VH, respectively. The bar plot shows the number of isolated with (dark grey) and without (no colour) *vanA/vanB* genes in *E. faecalis* and *E. faecium* from both hospitals. *E. faecium* carried vancomycin resistance genes significantly more frequently than *E. faecalis* across both hospitals. A higher proportion of *van*-gene-positive *E. faecium* was observed in REUH compared to VH, while *E. faecalis* remained largely *van*-gene-negative in both settings.

The bar plot (Figure 1) depicts the numbers of positive and negative cases for vancomycin (*vanA* and *vanB*) resistance genes in *E. faecalis* and *E. faecium* isolates from two Latvian hospitals (REUH and VH). REUH has significantly more vancomycin-resistant *E. faecium* than VH (*p*-value = 0.007), and a similar trend is observed for *E. faecalis* (*p*-value = 0.070). *E. faecium* carried vancomycin resistance genes significantly more frequently than *E. faecalis* (*p*-value < 0.001).

A detailed list of detected *vanA* and *vanB* genes and overall resistance prevalence in *E. faecalis* and *E. faecium* is summarised in Table 5.

### 2.4. Results of MLST and cgMLST

*E. faecium*

**Figure 2:**
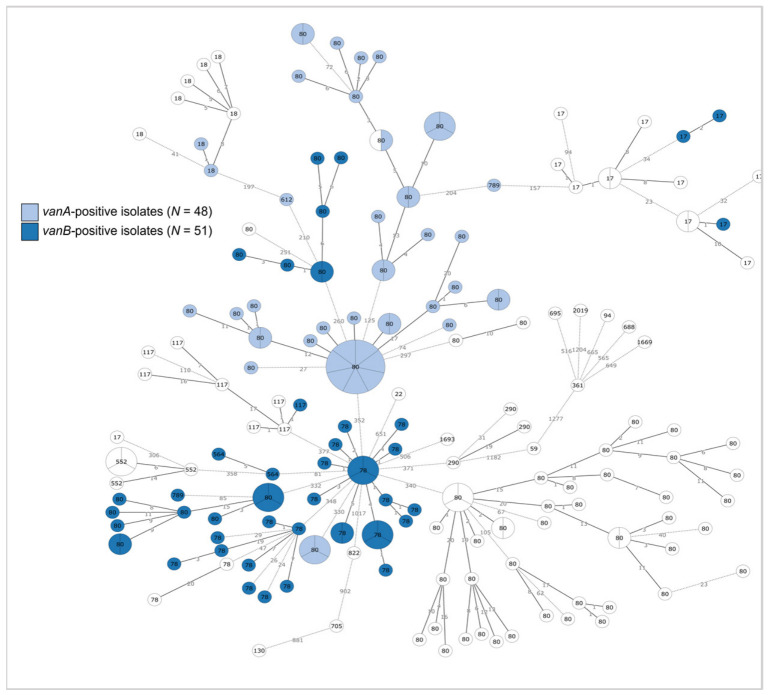
cgMLST minimum spanning tree of *E. faecium* isolates. Nodes are labelled with ST identifiers and colour-coded according to the presence of *vanA* or *vanB* genes. Branch lengths are logarithmically scaled. The tree shows genetic relatedness of *E. faecium* isolates based on cgMLST, where node size reflects cluster size and colours indicate vancomycin resistance genes (*vanA*, *vanB*, or none). Two dominant lineages, ST80 and ST78, account for most isolates. ST80 includes both *vanA*- and *vanB*-positive and susceptible clusters. ST78 is predominantly composed of *vanB*-positive clusters.

Using MLST analysis, we identified 22 distinct sequence types (STs). Several outbreak-relevant clonal clusters were detected among *E. faecium* isolates, defined at the level of cgMLST complex types (CTs) within MLST sequence types. Two major MLST lineages, ST80 and ST78, formed epidemiologically relevant clusters in this study (Figure 2).

**Figure 3:**
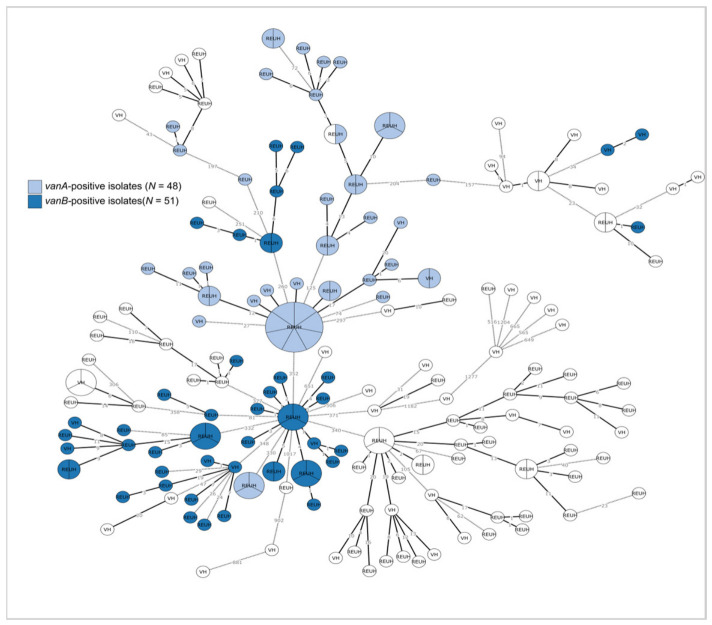
cgMLST minimum spanning tree of *E. faecium* isolates. Nodes are labelled with the hospital of origin and colour-coded according to the presence of *vanA* or *vanB* genes. Branch lengths are logarithmically scaled. The tree demonstrates that major lineages (ST80 and ST78) are distributed across both hospitals rather than confined to a single institution.

**Figure 4:**
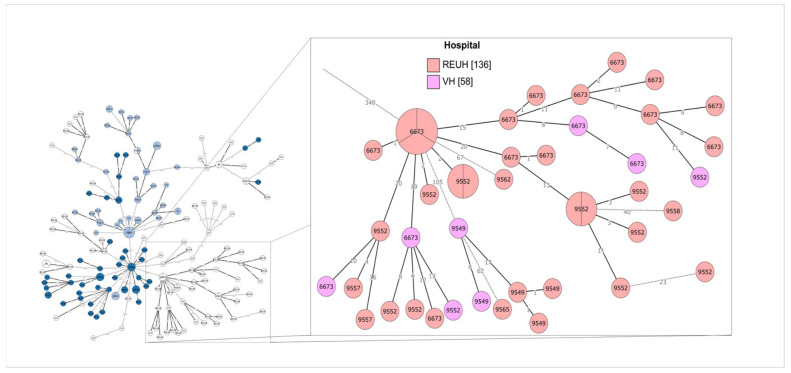
A zoomed-in view of the cgMLST minimum spanning tree focusing on vancomycin-susceptible *E. faecium* population structure. Nodes are labelled with cgMLST complex type identifiers and colour-coded according to the hospital of origin. Branch lengths in the cgMLST minimum spanning tree are logarithmically scaled. The susceptible population shows a structured clustering pattern, mainly across REUH, with some clusters spreading across both institutions.

Within ST80, four distinct cgMLST CTs were identified. CT2046 (*N* = 22) represented a *vanA*-positive outbreak cluster, more frequently observed in VH (*N* = 13) but also present in REUH (*N* = 9). CT2579 (*N* = 10) carried the *vanB* gene and was detected predominantly in REUH (*N* = 8), with two cases identified in VH. CT9552 (*N* = 16) constituted a mixed cluster, predominantly composed of *vanA*-positive isolates (15/16), with one vancomycin-susceptible isolate (Figure 3). In addition, CT6673 (*N* = 19) represented a vancomycin-susceptible *E. faecium* outbreak cluster, observed mainly in REUH (*N* = 15) compared with VH (*N* = 4) (Figure 4).

Within ST78, two major *vanB*-positive cgMLST clusters were identified. CT9553 (*N* = 18) constituted the largest outbreak cluster and was detected primarily in REUH (*N* = 16), with two isolates identified in VH. CT9534 (*N* = 7) included five *vanB*-positive isolates from REUH and two from VH. A detailed overview of cgMLST clusters is presented in Table 6.

*E. faecalis*

**Figure 5:**
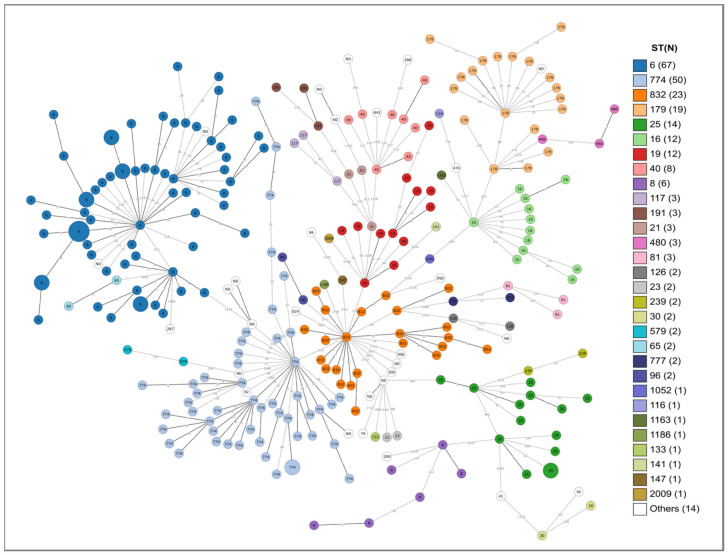
cgMLST-based population structure of *E. faecalis* coloured by MLST sequence types. Nodes represent cgMLST CTs and colours indicate MLST STs. Node size reflects the number of isolates, and branch lengths correspond to allelic differences (logarithmic scale). The tree demonstrates the pattern of genetically heterogeneous and polyclonal population structure, with ST6, ST774, and ST832 representing the dominant lineages, each comprising multiple CTs distributed across the tree.

cgMLST analysis of *E. faecalis* isolates revealed a genetically heterogeneous population comprising multiple STs and cgMLST CTs. Most isolates belonged to three predominant STs—ST6, ST774, and ST832—each represented by multiple CTs (Figure 5). Among these lineages, ST6 was the most widely distributed across both hospitals and comprised multiple cgMLST CTs.

*VanB*-positive *E. faecalis* was detected across multiple cgMLST complex types within the ST6 clonal lineage. In total, *vanB*-positive isolates were identified across 15 cgMLST CTs, including one isolate without an assigned MLST sequence type (Table 7). The majority of vancomycin-resistant *E. faecalis* isolates originated from REUH (*N* = 13), while five isolates were detected in VH.

**Figure 6:**
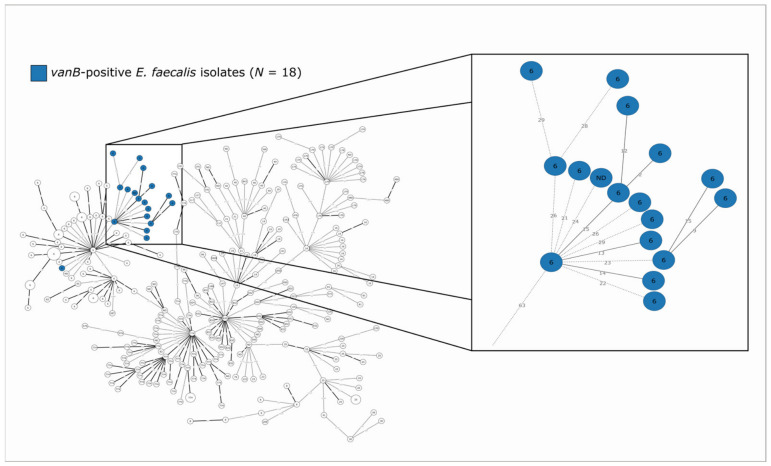
Distribution of *vanB*-positive ST6 *E. faecalis* isolates across both healthcare settings. The figure presents a focused view of the ST6 lineage, highlighting the *vanB*-positive isolates (blue) within the cgMLST minimum spanning tree. Branch lengths of the cgMLST minimum spanning tree, with labels indicating the hospital of origin (REUH or VH), are logarithmically scaled. *VanB*-positive isolates are distributed across multiple CTs within ST6 rather than forming a single tight cluster. These isolates were detected in both hospitals, with a higher number originating from REUH.

Within ST832, several closely related vancomycin-susceptible *E. faecalis* isolates clustered within individual cgMLST CTs and were detected in both hospitals across multiple time points during the study period. Similarly, ST774 comprised multiple CTs that were distributed throughout the entire study period and identified in both hospitals. The majority of *vanB*-resistant *E. faecalis* isolates were detected in REUH (Figure 6). Several vancomycin-susceptible CTs appeared to be hospital-specific, whereas others were observed in both hospitals.

No *vanA*-positive isolates were identified among *E. faecalis* isolates.

MLST analysis revealed that *E. faecalis* isolates predominantly belonged to ST6, ST774, ST832, ST179, and ST25. One isolate from REUH belonged to cgMLST CT4273 with no listed MLST ST.

*Vancomycin-Variable Enterococcus (VVE)*

Three isolates (0.62% of all analysed isolates) were identified as vancomycin-variable enterococci (VVE). All three isolates were *E. faecium* carrying the *vanA* gene while remaining phenotypically susceptible to vancomycin. All VVE isolates originated from REUH and accounted for 1.03% of all *Enterococcus* spp. isolates obtained at REUH.

MLST analysis revealed that all three VVE isolates belonged to ST80, and they shared the same cgMLST complex type (CT7202). The isolates were recovered from three different patients admitted to different departments of REUH; however, they were detected within a narrow time frame (25 March–11 April 2024).

Three isolates were classified as vancomycin-variable *enterococci* (VVE). Genomic analysis demonstrated that all three isolates harboured *vanH*, *vanX*, *vanY* and *vanZ*, but lacked the *vanS* and *vanR* genes within the *vanA* gene cluster. Phenotypically, all were susceptible to vancomycin (inhibition zone 16–18 mm).

SNP analysis supported the possibility of epidemiological linkage: post-recombination filtering showed a maximum pairwise distance of ≤9 SNPs (range 2–9 SNPs) among the isolates of this cluster, as detailed in Table 8. Isolates No. 214-2024 and No. 199-2024 exhibited a pairwise distance of only two SNPs—the tightest linkage observed.

## 3. Discussion

Our whole-genome sequencing analysis of 482 *Enterococcus* spp. isolates collected over a four-year period from two tertiary hospitals in Latvia (REUH, 61.1%; VH, 38.9%) represents a comprehensive genomic extension of our initial 143-isolate WGS pilot study (Labecka et al., 2024) [36]. This systematic progression confirms pronounced, species-specific vancomycin-resistant *enterococcus* (VRE) epidemiological patterns observed across Europe [17,23]. In our cohort, *E. faecium* accounted for 79.20% of VRE cases, predominantly driven by hospital-adapted ST80 and ST78 lineages, whereas *E. faecalis*—despite representing 56.64% of all Enterococcus isolates—exhibited a largely polyclonal endemic population with a comparatively modest VRE burden (14.40% of all VRE isolates). This mirrors the established dominance of *E. faecium* in hospital-associated VRE epidemiology reported in Germany and other European countries [17,40].

Contrary to the historical predominance of *vanA* in Europe, *vanB* was the dominant resistance determinant in our study (54.40%, 68/125), exceeding *vanA* (39.20%, 49/125). Both resistance genes were distributed across species (*E. faecium*, 75%; *E. faecalis*, 25%), whereas *vanA* remained almost exclusively associated with *E. faecium* (97.96%) [24,38,41]. This pattern aligns with recent Northern European trends, including Denmark’s shift from *vanA* dominance (71% in 2015) to *vanB* predominance (50% in 2022) [24,33], and reports from German university hospitals where 77.1% of 363 VRE isolates (2016–2020) carried *vanB*, reflecting endemic circulation of *vanB*-positive *E. faecium* lineages in hospital settings [13,16].

Institution-specific differences were evident, with REUH showing predominance of *vanB*, whereas VH remained largely *vanA*-dominated. Such regional heterogeneity likely reflects differences in antimicrobial selection pressure, historical introduction of resistance determinants, and local transmission dynamics [42,43]. Notably, this divergence persists despite regular patient exchange between hospitals under the national hospitalisation framework, suggesting persistent institution-specific epidemiological patterns of VRE.

Intrinsic *vanC*-mediated resistance (6.40%), restricted to *E. gallinarum* and *E. casseliflavus*, was also confirmed. As expected, these isolates represented a minor proportion of the cohort [19,20]. Collectively, these findings reinforce the notion that clinically relevant vancomycin resistance in Latvian hospitals, as elsewhere in Europe, is primarily driven by acquired resistance mechanisms in *E. faecium* [23,40,44,45].

High-resolution cgMLST analysis identified 22 *E. faecium* STs, with outbreak-associated transmission confined predominantly to ST80 (67 isolates across four cgMLST CTs) and ST78 (25 isolates across two CTs), both belonging to the archetypal hospital-adapted CC17 lineage. ST80 demonstrated remarkable genetic and phenotypic plasticity, encompassing CT2046 (*vanA*; *N* = 22, VH-associated outbreak), CT2579 (*vanB*; *N* = 10, predominantly REUH), CT9552 (mixed phenotype; *N* = 16, 15/16 *vanA*-positive), and CT6673 (vancomycin-susceptible; *N* = 19, REUH). This resistance heterogeneity within a historically dominant European hospital clone mirrors observations from Germany and Denmark, including persistent ST80 lineages and the recent emergence of *vanB*-positive variants [24,25,26]. To our knowledge, this study provides the first Baltic WGS-based baseline documenting co-circulation of ST80-CT2046 (*vanA*) and ST80-CT2579 (*vanB*) within the broader Northern European CC17 epidemiological context.

ST78 exhibited greater homogeneity, with stable *vanB* integration observed across CT9553 (*N* = 18, indicating inter-hospital transmission) and CT9534 (*N* = 7), supporting the capacity of this lineage for clonal dissemination between institutions [45].

In contrast, cgMLST analysis of *E. faecalis* revealed a fundamentally different epidemiological pattern. High genetic diversity was observed across ST6, ST774, and ST832, with *vanB* dispersed across 15 distinct ST6-associated CTs (*N* = 18). This polyclonal distribution suggests endemic circulation rather than outbreak-driven hospital transmission, consistent with the substantially lower VRE burden in *E. faecalis* (6.6%) compared with *E. faecium* (51%) reported previously [36]. The predominance of *vanB*-positive isolates at REUH (13/18) indicates institution-specific amplification, whereas shared vancomycin-susceptible CTs between hospitals reflect baseline endemicity. The absence of *vanA* clusters in *E. faecalis* is consistent with previous reports indicating that *vanA*-mediated resistance is predominantly associated with *E. faecium* rather than *E. faecalis* [22,23].

The identification of three vancomycin-variable *E. faecium* isolates (0.62%; ST80/CT7202) represents, to our knowledge, the first documented VVE cases reported in Latvia. All isolates were recovered from REUH within a narrow temporal window (March–April 2024) and exhibited phenotypic susceptibility to vancomycin despite harbouring a *vanA*-type gene cluster comprising *vanHXYZ* but lacking the sensory gene *vanS* and the regulatory gene *vanR*. This genomic configuration may contribute to the observed genotype–phenotype discordance and is consistent with previous reports of VVE outbreaks in Denmark and other European countries [24,32,39,46]. As inducible *vanA*-type resistance fundamentally relies on *vanR*-mediated transcriptional activation, the absence of *vanR/vanS* in these isolates may reduce the likelihood of *vanA*-mediated inducible resistance expression [30,34,35]. However, alternative regulatory or structural explanations cannot be excluded, and the broader genetic context, including associated transposons and plasmid structures, was beyond the scope of this study.

The close temporal–spatial clustering across three REUH departments suggests possible cryptic transmission not detectable by routine phenotypic screening alone. Additionally, the mixed-phenotype CT9552 cluster (15/16 *vanA*-positive) further indicates potential regulatory instability within the ST80 lineage [32,33]. Core genome SNP (cgSNP) analysis demonstrated a high degree of genetic linkage, with a post-recombination filtering maximum distance of ≤9 SNPs. Isolate No. 199 and No. 214 were collected within a six-day interval, exhibiting only two cgSNPs—the tightest linkage observed—suggesting a close epidemiological link and probable transmission. Subsequent dissemination to isolate No. 243, differing by four SNPs from the index case, further supports short-term transmission dynamics. This 17-day dissemination was not detectable using routine phenotypic diagnostics.

From a clinical perspective, VVE isolates may represent a diagnostically challenging subset of *enterococci* that could be misclassified as vancomycin-susceptible during routine susceptibility testing despite harbouring *van* gene clusters (“silent” *van* genes). Although the absence of regulatory elements likely limits inducible resistance expression, the presence of structural *van* genes suggests retained genetic potential for resistance, which may result in delayed initiation of effective therapy and increased risk of adverse clinical outcomes [29,47,48].

The advantages of cgMLST were clearly demonstrated in this study. Seven transmission clusters, including CT2046, CT9552, and CT6673, were identified, which would not have been resolved using MLST alone. Resistance profiles were CT-specific within STs, with ST80 encompassing the full spectrum of phenotypes (*vanA*, *vanB*, mixed, and susceptible), whereas ST78 was uniformly associated with *vanB*. This level of resolution supports cgMLST as an essential tool for modern infection control and surveillance, surpassing traditional typing approaches [24,27,28,49].

Overall, this study establishes a national reference dataset for VRE and VVE in Latvia within the broader Northern European context, providing methodological continuity from phenotypic to high-resolution genomic surveillance.

Several limitations should be acknowledged. The study was restricted to two tertiary hospitals, which may limit the complete representation of national epidemiological trends. In addition, sampling intensity varied across the study period, influenced in part by the redistribution of laboratory workload during the COVID-19 pandemic. Consequently, some aspects of inter-hospital transmission dynamics may be captured incompletely. Nevertheless, integrating longitudinal phenotypic data with high-resolution genomic analysis provides a robust framework for characterising vancomycin resistance epidemiology at local, national, and regional levels.

## 4. Materials and Methods

### 4.1. Study Design and Sampling

In our study, *Enterococcus* spp. isolates obtained from routine clinical diagnostics were collected for genomic investigation of genetic variability at two tertiary-level hospitals in Latvia: Riga East University Hospital (REUH) and Vidzeme Hospital (VH). Clinical specimens from adult patients were collected from 2021 to 2024 and routinely processed in hospital microbiology laboratories according to local practice. The study was based on a longitudinal collection of *Enterococcus* spp. isolates submitted to the Institute of Food Safety, Animal Health and Environment “BIOR” from routine clinical diagnostics at both hospitals over the study period. Confirmatory species identification was performed by MALDI-TOF MS (Bruker, Ettlingen, Germany), and isolates were stored in the bacterial cryobank. Isolates of insufficient quality, including those with contamination or unidentifiable species, were excluded from further study, and a total of 532 *Enterococcus* isolates were eligible for genetic testing. For the final analysis, only isolates from hospitalised patients that passed sequencing quality control were included. The study was approved by the Ethics Committee of Riga Stradiņš University, 16 Dzirciema Str., LV-1007, Riga, Latvia, approval no. 6-1/09/11, 10 September 2020.

### 4.2. Microbiological Testing of Samples

In both hospital microbiology laboratories, isolates were cultured on 5% sheep blood agar plates (Columbia Blood Agar Base, HiMedia Laboratories, Thane, India), supplemented with defibrinated sheep blood (TCS Biosciences Ltd., Buckingham, UK). In REUH, species identification was performed by matrix-assisted laser desorption/ionisation time-of-flight (MALDI-TOF MS) (Bruker, Ettlingen, Germany), whereas VH identification was conducted using the Vitek MS system (bioMérieux, Craponne, France). In REUH, phenotypic antimicrobial susceptibility testing was performed using the disc diffusion method (Liofilchem, Roseto degli Abruzzi, Italy) and interpreted according to the European Committee on Antimicrobial Susceptibility Testing (EUCAST) criteria in effect at the time of testing. In VH, antimicrobial susceptibility testing for *Enterococcus* spp. was performed using the Vitek-2 system (BioMérieux, Craponne, France), with GP identification cards and AST 643 susceptibility cards. The results were interpreted using EUCAST breakpoints applicable at the time of analysis.

### 4.3. Extraction of DNA, Whole-Genome Sequencing (WGS)

Species identification of the isolates was reconfirmed by MALDI-TOF MS (Bruker, Ettlingen, Germany). Genomic DNA was extracted using the NucleoSpin Tissue kit (Macherey-Nagel, Düren, Germany) with lysozyme-assisted cell wall lysis according to the manufacturer’s instructions. DNA purity and concentration were assessed using NanoDrop and Qubit instruments, respectively (ThermoFisher Scientific, Landsmeer, The Netherlands). Whole-genome sequencing libraries were prepared using the Illumina DNA Prep kit and quality-checked by capillary gel electrophoresis (QIAxcel Advanced Instrument, QIAGEN, Venlo, Limburg, The Netherlands), followed by paired-end sequencing either on the Illumina MiSeq or NextSeq 2000 platform (Illumina, San Diego, CA, USA).

### 4.4. Genome Assembly, Detection of Resistance Genes, and Core Genome Sequence Typing

Raw sequencing data were quality-controlled, trimmed, and assembled into de novo genomes as described previously [36,50].

Genome quality control was conducted to exclude assemblies that could compromise downstream analyses. Assemblies were assessed based on taxonomic assignment, GC content, genome length, N50 value, and sequencing depth. Taxonomic affiliation was verified in silico using a metagenomic classifier (kraken2) to identify potential misidentification or sample contamination. Assemblies were considered acceptable if GC content ranged between 36% and 41%, genome length ranged between 2.70 and 3.85 Mb, N50 ≥ 20,000 bp, and average sequencing depth ≥25×. Based on these criteria, 19 samples out of 532 were excluded due to insufficient sequencing quality.

The genome assemblies were screened for the presence of antimicrobial resistance determinants using the ResFinder tool (v4.7.2) and the ResFinder, PointFinder, and DisinFinder databases (versions of 10 April 2025, 8 August 2025 and 31 August 2025, respectively) [51,52].

Core genome multilocus sequence typing (cgMLST) was performed using SeqSphere+ v10.5.4 software (Ridom GmbH, Münster, Germany) to assess genetic relatedness, investigate transmission pathways, and support outbreak detection. For *E. faecalis*, the cgMLST scheme by Neumann et al. (2019) [53] was used, but for *E. faecium*—the scheme by de Been et al. (2015) [49]. cgMLST results were visualised and analysed using a minimum spanning tree approach implemented in GrapeTree (v1.5.0) software with the MSTreeV2 algorithm [54].

MLST sequence types (STs) were used to observe long-term clonal lineages, whereas cgMLST complex types (CTs) were used to identify potential transmission clusters and outbreak episodes. cgMLST clusters were defined according to thresholds of 7 or 20 allelic differences for *E. faecalis* and *E. faecium*, respectively.

For specific isolate clusters of interest, read mapping against the reference genome CP012430.1 was performed by the nf-core/bactmap pipeline (v1.0.0) [55] with recombination removal enabled, and a SNP distance matrix was calculated using the snp-dists tool (https://github.com/tseemann/snp-dists, accessed on 5 January 2026).

### 4.5. Statistical Analysis

Descriptive and inferential statistical analyses were performed using the R statistical software (v4.5.1). Qualitative variables were summarised as absolute frequencies (N) and percentages (%). Comparisons between independent categorical variables were conducted using Pearson’s chi-square (χ^2^) test when all expected cell frequencies were ≥5; otherwise, Fisher’s exact test was applied. Quantitative variables were described using the median (Md) and interquartile range (Q1–Q3), and differences between two independent groups were assessed using the Mann–Whitney U test.

Differences in the distribution of vancomycin resistance genes (*vanA* and *vanB*) between hospitals were evaluated separately for each bacterial species using the likelihood-ratio G-test (DescTools package, v0.99.59). Comparisons between species, while controlling for hospital as a stratification variable, were performed using the Mantel–Haenszel chi-squared test with continuity correction. Figures were created using the ggplot2 package [56]. The level of statistical significance was set at α = 0.05, and two-sided *p*-values < 0.05 were considered statistically significant.

## 5. Conclusions

Vancomycin resistance in *Enterococcus* spp. was predominantly associated with *E. faecium*, with the *vanB* gene as the predominant resistance determinant in both hospitals. This finding contrasts with earlier *vanA* predominance reported in Europe but aligns with more recent regional trends, with inter-hospital differences suggesting institution-specific epidemiological patterns.

WGS/cgMLST analysis identified hospital-adapted *E. faecium* lineages (ST80/ST78) dominating the population structure, characterised by clonal expansion and indicative evidence of inter-hospital dissemination, alongside a more polyclonal distribution of *E. faecalis*.

The identification of vancomycin-variable *E. faecium* (VVE) harbouring incomplete *vanA* clusters lacking sensory/regulatory (*vanS*/*vanR*) elements highlights the clinically relevant genotype–phenotype discordance and suggests potential cryptic dissemination of vancomycin resistance determinants. These findings underscore the importance of integrating genomic surveillance with routine phenotypic testing to improve detection of “silent” *van* genes, infection control, and antimicrobial therapy guidance.

## Figures and Tables

**Table 1:** Age distribution of patients by hospital and gender.

Characteristic	REUH Women *N* = 101; 43.9%	REUH Men *N* = 129; 56.1%	VH Women *N* = 83; 48.3%	VH Men *N* = 89; 51.7%	*p*-Value *
Median age, years (Q1–Q3)	73.0 (64.0–82.0)	70.0 (57.8–79.0)	75.0 (65.0–80.0)	69.0 (58.2–79.0)	<0.01

* *p*-value for comparison between hospitals within a gender.

**Table 2:** Distribution of biological specimen types stratified by each hospital.

Specimen	Total (*N* = 482) *N* (%)	REUH (*N* = 291) *N* (%)	VH (*N* = 191) *N* (%)
Blood	44 (9.13)	32 (11.00)	12 (6.28)
Lower respiratory tract secretions	22 (4.56)	10 (3.44)	12 (6.28)
Faeces	42 (8.71)	42 (14.43)	0 (0)
Gynaecological specimens *	6 (1.24)	3 (1.03)	3 (1.57)
Soft tissue specimens	105 (21.78)	62 (21.31)	43 (22.51)
Sterile body fluids **	22 (4.56)	20 (6.87)	2 (1.05)
Urine	240 (49.79)	121 (41.58)	119 (62.30)
Urogenital specimens	1 (0.21)	1 (0.34)	0 (0)

* Gynaecological specimens comprised specimens obtained from the female genital tract and related obstetric/gynaecological procedures, including swab specimens, intrauterine samples, and gynaecological surgical materials. ** Sterile body fluids comprised specimens classified in routine diagnostics as fluids, cavity contents, or punctates obtained from normally sterile body sites.

**Table 3:** Distribution of *Enterococcus* species stratified by each hospital.

Species	Total (*N* = 482) *N* (%)	REUH (*N* = 291) *N* (%)	VH (*N* = 191) *N* (%)
*Enterococcus faecalis*	273 (56.64)	142 (48.80)	131 (68.59)
*Enterococcus faecium*	194 (40.25)	136 (46.74)	58 (30.37)
*Enterococcus gallinarum*	6 (1.24)	6 (2.06)	0 (0)
*Enterococcus avium*	3 (0.62)	2 (0.69)	1 (0.52)
*Enterococcus casseliflavus*	2 (0.41)	2 (0.69)	0 (0)
*Enterococcus durans*	2 (0.41)	2 (0.69)	0 (0)
*Enterococcus hirae*	1 (0.21)	0 (0)	1 (0.52)
*Enterococcus raffinosus*	1 (0.21)	1 (0.34)	0 (0)

**Table 4:** Distribution of *Enterococcus* species according to biological specimen type.

Specimen	*E. faecalis* (*N* = 273)	*E. faecium* (*N* = 194)	*E. gallinarum* (*N* = 6)	*E. avium* (*N* = 3)	*E. casseliflavus* (*N* = 2)	*E. durans* (*N* = 2)	*E. hirae* (*N* = 1)	*E. raffinosus* (*N* = 1)
Blood	19	24	0	1	0	0	0	0
Lower respiratory tract secretions	13	9	0	0	0	0	0	0
Faeces	5	37	0	0	0	0	0	0
Gynaecological specimens	4	2	0	0	0	0	0	0
Soft tissue specimens	57	42	2	1	1	2	0	0
Sterile body fluids	6	14	1	0	1	0	0	0
Urine	168	66	3	1	0	0	1	1
Urogenital specimens	1	0	0	0	0	0	0	0

**Table 5:** Prevalence of *vanA* and *vanB* genes among *Enterococcus* spp. isolates.

Species	Total Resistant Isolates, *N*	*van* Genes	Total, *N*	REUH, *N*	VH, *N*	Resistance Prevalence (Overall) *, %	Resistance Prevalence (REUH) *, %	Resistance Prevalence (VH), %
*E. faecalis*	18	*vanA*	0	0	0	6.59	9.16	3.82
		*vanB*	18	13	5			
*E. faecium*	99	*vanA*	48	35	13	51.03	57.35	36.21
		*vanB*	51	43	8			

* Prevalence of resistance calculated by using the total number of Enterococcus isolates of respective species overall and per hospital denominator.

**Table 6:** Overview of cgMLST clusters in *E. faecium*.

MLST ST	cgMLST CT	Vancomycin Resistance Determinant	Total (*N*)	REUH (*N*)	VH (*N*)
80	2046	*vanA*	22	9	13
80	6673	none (vancomycin susceptible)	19	15	4
80	2579	*vanB*	10	8	2
80	9552	*vanA* (15/16) + none (susceptible; 1/16)	16	15 + 1	0
78	9553	*vanB*	18	16	2
78	9534	*vanB*	7	5	2

**Table 7:** STs and CTs with listed number of *vanB*-positive isolates in *E. faecalis*.

MLST ST	cgMLST CT	*vanB* Gene; *N* = 18 (*N*)	REUH; *N* = 13 (*N*)	VH; *N* = 5 (*N*)
6	3061	2	2	0
6	4136	1	1	0
6	4137	2	0	2
6	4142	1	1	0
6	4148	1	1	0
6	4154	1	0	1
6	4203	1	0	1
6	4218	**1**	1	0
6	4266	2	2	0
6	4307	1	1	0
6	4312	1	0	1
6	4315	1	1	0
6	4322	**1**	1	0
6	4331	**1**	1	0
not listed	4273	1	1	0

**Table 8:** SNP distance matrix of vancomycin-variable *E. faecium* CT7202 transmission cluster (REUH, 2024) before accounting for recombination.

Date of Sampling	Snp-Dists 1.2.0	Isolate No. 243-2024	Isolate No. 214-2024	Isolate No. 199-2024	CP012430.1 (ref)
11 April 2024	Isolate No. 243-2024	0	169	18	3851
31 March 2024	Isolate No. 214-2024	169	0	271	4521
25 March 2024	Isolate No. 199-2024	18	271	0	4069
-	CP012430.1 (ref)	3851	4521	4069	0

## Data Availability

Research data can be obtained upon request.

## Abbreviations

The following abbreviations are used in this manuscript:

AMR	Antimicrobial resistance
AST-GP cards	Antimicrobial susceptibility testing Gram-Positive cards
BSIs	Bloodstream infections
CA	California
CC	Clonal complex
CDC	Centers for Disease Control and Prevention
cgMLST	Core genome multilocus sequence typing
cgSNP	Core genome single nucleotide polymorphism
CT	Complex type
DNA	Deoxyribonucleic acid
ECDC	European Centre for Disease Prevention and Control
ESKAPE	*Enterococcus faecium*, *Staphylococcus aureus*, *Klebsiella pneumoniae*, *Acinetobacter baumannii*, *Pseudomonas aeruginosa*, *Enterobacter* spp.
EUCAST	European Committee on Antimicrobial Susceptibility Testing
GC	Guanine Cytosine bases
MALDI-TOF MS	Matrix-assisted laser desorption/ionisation time-of-flight mass spectrometry
MLST	Multilocus sequence typing
REUH	Riga East University Hospital
SNP	Single nucleotide polymorphism
ST	Sequence type
spp.	Species
US/USA	United States of America
UTIs	Urinary tract infections
VH	Vidzeme Hospital
VRE	Vancomycin-resistant *Enterococci*
VVE	Vancomycin-variable *Enterococci*
WGS	Whole-genome sequencing
WHO	World Health Organisation

## References

[1] Naghavi M., Vollset S.E., Ikuta K.S., Swetschinski L.R., Gray A.P., Wool E.E., Aguilar G.R., Mestrovic T., Smith G., Han C. (2024). Global Burden of Bacterial Antimicrobial Resistance 1990–2021: A Systematic Analysis with Forecasts to 2050. Lancet.

[2] Murray C.J., Ikuta K.S., Sharara F., Swetschinski L., Aguilar G.R., Gray A., Han C., Bisignano C., Rao P., Wool E. (2022). Global Burden of Bacterial Antimicrobial Resistance in 2019: A Systematic Analysis. Lancet.

[3] WHO Regional Office for Europe and European Centre for Disease Prevention and Control (2025). Surveillance of Antimicrobial Resistance in Europe. 2024 Data: Executive Summary.

[4] Ramos S., Silva V., Dapkevicius M.d.L.E., Igrejas G., Poeta P. (2020). *Enterococci*, from Harmless Bacteria to a Pathogen. Microorganisms.

[5] Lebreton F., Manson A.L., Saavedra J.T., Straub T.J., Earl A.M., Gilmore M.S. (2017). Tracing the *Enterococci* from Palaeozoic Origins to the Hospital. Cell.

[6] Pandova M., Kizheva Y., Tsenova M., Rusinova M., Borisova T., Hristova P. (2024). Pathogenic Potential and Antibiotic Susceptibility: A Comprehensive Study of *Enterococci* from Different Ecological Settings. Pathogens.

[7] Krawczyk B., Wityk P., Gałęcka M., Michalik M. (2021). The Many Faces of *Enterococcus* spp.—Commensal, Probiotic and Opportunistic Pathogen. Microorganisms.

[8] Codelia-Anjum A., Lerner L.B., Elterman L.B., Zorn K.C., Bhojani N., Chughtai B. (2023). *Enterococcal* Urinary Tract Infections: A Review of the Pathogenicity, Epidemiology, and Treatment. Antibiotics.

[9] Zhou X., Willems R.J.L., Friedrich A.W., Rossen J.W.A., Bathoorn E. (2020). *Enterococcus faecium*: From Microbiological Insights to Practical Recommendations for Infection Control and Diagnostics. Antimicrob. Resist. Infect. Control.

[10] Miller W.R., Arias C.A. (2024). ESKAPE Pathogens: Antimicrobial Resistance, Epidemiology, Clinical Impact and Therapeutics. Nat. Rev. Microbiol..

[11] Arias C.A., Murray B.E. (2012). The Rise of the *Enterococcus*: Beyond Vancomycin Resistance. Nat. Rev. Microbiol..

[12] Ayobami O., Willrich N., Reuss A., Eckmanns T., Markwart R. (2020). The Ongoing Challenge of Vancomycin-Resistant *Enterococcus faecium* and *Enterococcus faecalis* in Europe: An Epidemiological Analysis of Bloodstream Infections. Emerg. Microbes Infect..

[13] Hornuss D., Göpel S., Walker S.V., Tobys D., Häcker G., Seifert H., Higgins P.G., Xanthopoulou K., Gladstone B.P., Cattaneo C. (2024). Epidemiological Tends and Susceptibility Patterns of Bloodstream Infections Caused by *Enterococcus* spp. in Six German University Hospitals: A Prospectively Evaluated Multicentre Cohort Study from 2016 to 2020 of the R-Net Study Group. Infection.

[14] Ghazvinian M., Asgharzadeh M.S., Gholami M., Gholami A.S., Amiri E., Goli H.R. (2024). Antimicrobial Resistance Patterns, Virulence Genes, And Biofilm Formation in *Enterococci* Strains Collected from Different Sources. BMC Infect. Dis..

[15] Khalil M.A., Alorabi J.A., Al-Otaibi L.M., Ali S.S., Elsilk S.E. (2023). Antibiotic Resistance and Biofilm Formation in *Enterococcus* spp. Isolated from Urinary Tract Infections. Pathogens.

[16] Cimen C., Berends M.S., Bathoorn E., Lokate M., Voss A., Friedrich A.W., Glasner C., Hamprecht A. (2023). Vancomycin-Resistant *Enterococci* (VRE) in Hospital Settings Across European Borders: A Scoping Review Comparing the Epidemiology in the Netherlands and Germany. Antimicrob. Resist. Infect. Control.

[17] Puchter L., Chaberny I.F., Schwab F., Vonberg R.P., Bange F.C., Ebadi E. (2018). Economic Burden of Nosocomial Infections Caused by Vancomycin-Resistant *Enterococci*. Antimicrob. Resist. Infect. Control.

[18] Bager P., Kähler J., Andersson M., Holzknecht B.J., Hansen S.G.K., Schønning K., Nielsen K.L., Koch K., Pinholt M., Voldstedlund M. (2024). Comparison of Morbidity and Mortality After Bloodstream Infection with Vancomycin-Resistant Versus -Susceptible *Enterococcus faecium*: A Nationwide Cohort Study in Denmark, 2010–2019. Emerg. Microbes Infect..

[19] Courvalin P. (2006). Vancomycin Resistance in Gram-Positive *Cocci*. Clin. Infect. Dis..

[20] Lebreton F., Depardieu F., Bourdon N., Fines-Guyon M., Berger P., Camiade S., Leclercq R., Courvalin P., Cattoir V. (2011). D-Ala-D-Ser *VanN*-type Transferable Vancomycin Resistance in *Enterococcus faecium*. Antimicrob. Agents Chemother..

[21] Pfaller M.A., Cormican M., Flamm R.K., Mendes R.E., Jones R.N. (2019). Temporal and Geographic Variation in Antimicrobial Susceptibility and Resistance Patterns of *Enterococci*: Results from the SENTRY Antimicrobial Surveillance Program, 1997–2016. Open Forum Infect. Dis..

[22] Hollenbeck B.L., Rice L.B. (2012). Intrinsic and Acquired Resistance Mechanisms in *Enterococcus*. Virulence.

[23] Lu Z., Mclnnes R.S., Allen F., Gadar K., van Schaik W. (2025). Resistance to Last-Resort Antibiotics in *Enterococci*. FEMS Microbiol. Rev..

[24] Hammerum A.M., Karstensen K.T., Roer L., Kaya H., Lindegaard M., Porsbo L.J., Kjerulf A., Pinholt M., Holzknecht B.J., Worning P. (2024). Surveillance of Vancomycin-Resistant Enterococci Reveals Shift in Dominating Clusters from *VanA* to *VanB Enterococcus faecium* Clusters, Denmark, 2015 to 2022. Eurosurveillance.

[25] Knudsen M.J.S., Castruita J.A.S., Rubin I.M.C., Mollerup S., Johansen H.K., Marvig R.L., Nielsen K.L., Holzknecht B.J., Hoppe M., Kemp M. (2025). Genomic epidemiology of vancomycin-resistant *Enterococcus faecium* in Eastern Denmark from 2020 to 2022, and identification of *vanB* Tn*1549* insertion sites. Eur. J. Clin. Microbiol. Infect. Dis..

[26] Caplunik-Pratsch A., Kieninger B., Donauer V.A., Brauer J.M., Meier V.M.K., Seisenberger C., Rath A., Loibl D., Eichner A., Fritsch J. (2024). Introduction and Spread of Vancomycin-Resistant *Enterococcus faecium* (VREfm) at a German Tertiary Care Medical Center From 2004 Until 2010: A Retrospective Whole-Genome Sequencing (WGS) Study of the Molecular Epidemiology of VREfm. Antimicrob. Resist. Infect. Control.

[27] Park S., Ryoo N. (2024). Comparative Analysis of IR-Biotyper, MLST, cgMLST, and WGS for Clustering of Vancomycin-Resistant *Enterococcus faecium* in a Neonatal Intensive Care Unit. Microbiol. Spectr..

[28] Glasgow H.L., Zheng Y., Brazelton J.N., Tang L., Hayden R.T. (2025). Comparison of Core Genome Multi-Locus Sequencing Typing Pipelines for Hospital Outbreak Detection of Common Bacterial Pathogens. J. Clin. Microbiol..

[29] Hawkins M.R., Medvedeva N., Wang H., Banaei N., Holubar M.K. (2024). “Keeping us on our Toes”: A Review of What Clinicians Need to Know About Vancomycin-Variable *Enterococcus*. Antimicrob. Steward. Healthc. Epidemiol..

[30] von Wintersdorff C.J.H., Roelofsen M., Versteegh L., Benyahya Y., Jamin C., Mulder M., Bastiaens G.J.H., van Meer M.P.A., Flipse J. (2026). Vancomycin variable Enterococci in the Netherlands (2018–2023) and the mechanism of resistance induction. PLoS ONE.

[31] Coccitto S.N., Cinthi M., Simoni S., Pocognoli A., Zeni G., Mazzariol A., Morroni G., Mingoia M., Giovanetti E., Brenciani A. (2024). Genetic Analysis of Vancomycin-Variable *Enterococcus faecium* Clinical Isolates in Italy. Eur. J. Clin. Microbiol. Infect. Dis..

[32] Sivertsen A., Pedersen T., Larssen K.W., Bergh K., Rønning T.G., Radtke A., Hegstad K. (2016). A Silenced *VanA* Gene Cluster on a Transferable Plasmid Caused an Outbreak of Vancomycin-Variable *Enterococci*. Antimicrob. Agents Chemother..

[33] Hammerum A.M., Justesen U.S., Pinholt M., Roer L., Kaya H., Worning P., Nygaard S., Kemp M., Clausen M.E., Nielsen K.L. (2019). Surveillance of Vancomycin-Resistant Enterococci Reveals Shift in Dominating Clones and National Spread of a Vancomycin-Variable *VanA Enterococcus faecium* ST1421-CT1134 Clone, Denmark, 2015 to March 2019. Eurosurveillance.

[34] Phillips-Jones M., Channell G., Kelsall C., Hughes C.S., Ashcroft A.E., Patching S.G., Dinu V., Gillis R.B., Adams G.B., Harding S.E. (2017). Hydrodynamics of the *VanA*-type *VanS* histidine kinase: An extended solution conformation and first evidence for interactions with vancomycin. Sci. Rep..

[35] Almeida-Santos A.C., Novais C., Peixe L., Freitas A.R. (2025). Vancomycin-resistant Enterococcus faecium: A current perspective on resilience, adaptation, and the urgent need for novel strategies. J. Glob. Antimicrob. Resist..

[36] Labecka L., Ķibilds J., Cīrulis A., Čeirāne E.D., Zeltiņa I., Reinis A., Vilima B., Rudzīte D., Erts R., Mauliņa I. (2024). Evaluation of Antimicrobial Resistance in Clinical Isolates of *Enterococcus* spp. Obtained from Hospital Patients in Latvia. Medicina.

[37] Rotondo C., Antonelli V., Rossi A., D’Arezzo S., Selleri M., Properzi M., Turco S., Chillemi G., Dimartino V., Venditti C. (2025). Surveillance and Characterization of Vancomycin-Resistant and Vancomycin-Variable *Enterococci* in a Hospital Setting. Antibiotics.

[38] Pinholt M., Mollerup S., Boye K., Worning P., Holzknecht B.J., Nygaard S., Nielsen K.L., Hasman H., Roer L., Hammerum A.M. (2021). Investigation of the introduction and dissemination of *VanB Enterococcus faecium* in the Capital Region of Denmark and development of a rapid and accurate clone-specific *VanB E. faecium* PCR. J. Antimicrob. Chemother..

[39] Abdullah H.M., Marbjerg L.H., Andersen L., Hoegh S.V., Kemp M. (2022). A Simple and Rapid Low-Cost Procedure for Detection of Vancomycin-Resistance Genes in Enterococci Reveals an Outbreak of Vancomycin-Variable *Enterococcus faecium*. Diagnostics.

[40] van Hal S.J., Willems R.J.L., Gouliouris T., Ballard S.A., Coque T.M., Hammerum A.M., Hegstad K., Westh H.T., Howden B.P., Malhotra-Kumar S. (2021). The Global Dissemination of Hospital Clones of *Enterococcus faecium*. Genome Med..

[41] Egan S.A., Kavanagh N.L., Shore A.C., Mollerup S., Samaniego C.J.A., O’Connell B., McManus B.A., Brennan G.I., Pinholt M., Westh H. (2022). Genomic Analysis of 600 Vancomycin-Resistant *Enterococcus faecium* Reveals a High Prevalence of ST80 And Spread of Similar Vana Regions via IS1216E and Plasmid Transfer in Diverse Genetic Lineages in Ireland. J. Antimicrob. Chemother..

[42] Correa-Martínez C.L., Jurke A., Schmitz J., Schaumburg F., Kampmeier S., Mellmann A. (2022). Molecular Epidemiology of Vancomycin-Resistant *Enterococci* Bloodstream Infections in Germany: A Population-Based Prospective Longitudinal Study. Microorganisms.

[43] Almasaud L.D., Alkhulaifi M.M., Ghazawi A., Strepis N., Manzoor A., Senok A., Alajlan H.H., Almogbel M.S., Moradigaravand D., Khan M. (2025). High-Resolution Genomic and Molecular Characterization of Vancomycin-Resistant *Enterococci* from Hospitalized Patients in a Tertiary Care Center in Riyadh, Saudi Arabia. Sci. Rep..

[44] Zhou X., Chlebowicz M.A., Bathoorn E., Rosema S., Couto N., Lokate M., Arends J.P., Friedrich A.W., Rossen J.W.A. (2018). Elucidating Vancomycin-Resistant *Enterococcus faecium* Outbreaks: The Role of Clonal Spread and Movement of Mobile Genetic Elements. J. Antimicrob. Chemother..

[45] Brinkwirth S., Feig M., Noll I., Eckmanns T., Dörre A., Haller S., Willrich N. (2025). Changing Dynamics of Bloodstream Infections due to Methicillin-Resistant Staphylococcus Aureus and Vancomycin-Resistant *Enterococcus faecium* in Germany, 2017–2023: A Continued Burden of Disease Approach. Antimicrob. Resist. Infect. Control.

[46] Hansen T.A., Pedersen M.S., Nielsen L.G., Ma C.M.G., Søes L.M., Worning P., Østergaard C., Westh H., Pinholt M., Schønning K. (2018). Emergence of a Vancomycin-Variable *Enterococcus faecium* ST1421 Strain Containing a Deletion un *VanX*. *J. Antimicrob*. Chemother..

[47] McInnes R.S., Snaith A.E., Dunn S.J., Papangeli M., Hardy K.J., Hussain A., van Schaik W. (2024). Integration of *VanHAX* Downstream of a Ribosomal RNA Operon Restores Vancomycin Resistance in a Susceptible *Enterococcus faecium* Strain. npj Antimicrob. Resist..

[48] Coburn B., Low D.E., Patel S.N., Poutanen S.M., Shahinas D., Eshaghi A., Willey B.M., McGeer A. (2014). Vancomycin-Variable *Enterococcus* faecium: In Vivo Emergence of Vancomycin Resistance in a Vancomycin-Susceptible Isolate. J. Clin. Microbiol..

[49] De Been M., Pinholt M., Top J., Bletz S., Mellmann A., van Schaik W., Brouwer E., Rogers M., Kraat Y., Bonten M. (2015). Core Genome Multilocus Sequence Typing Scheme for High- Resolution Typing of *Enterococcus faecium*. J. Clin. Microbiol..

[50] Terentjeva M., Ķibilds J., Avsejenko J., Cīrulis A., Labecka L., Bērziņš A. (2024). Antimicrobial Resistance in *Enterococcus* spp. Isolates from Red Foxes (*Vulpes vulpes*) in Latvia. Antibiotics.

[51] Bortolaia V., Kaas R.S., Ruppe E., Roberts M.C., Schwarz S., Cattoir V., Philippon A., Allesoe R.L., Rebelo A.R., Florensa A.F. (2020). ResFinder 4.0 for Predictions of Phenotypes from Genotypes. J. Antimicrob. Chemother..

[52] Camacho C., Coulouris G., Avagyan V., Ma N., Papadopoulos J., Bealer K., Madden T.L. (2009). BLAST+: Architecture and applications. BMC Bioinform..

[53] Neumann B., Prior K., Bender J.K., Harmsen D., Klare I., Fuchs S., Bethe A., Zühlke D., Göhler A., Schwarz S. (2019). A Core Genome Multilocus Sequence Typing Scheme for *Enterococcus faecalis*. J. Clin. Microbiol..

[54] Zhou Z., Alikhan N.F., Sergeant M.J., Luhmann N., Vaz C., Francisco A.P., Carriço J.A., Achtman M. (2018). GrapeTree: Visualization of Core Genomic Relationships among 100,000 Bacterial Pathogens. Genome Res..

[55] Ewels P.A., Peltzer A., Fillinger S., Patel H., Alneberg J., Wilm A., Garcia M.U., Di Tommaso P., Nahnsen S. (2020). The nf-Core Framework for Community-Curated Bioinformatics Pipelines. Nat. Biotechnol..

[56] Wickham H. (2016). Ggplot2: Elegant Graphics for Data Analysis.

